# The Role of Twice-Daily N-acetylcysteine (NAC) 2400 mg in Smoking Cessation: A Randomized, Placebo-Controlled Trial in Indonesia

**DOI:** 10.7759/cureus.54322

**Published:** 2024-02-16

**Authors:** Annisa D Harlivasari, Agus D Susanto, Feni F Taufik, Tribowo T Ginting

**Affiliations:** 1 Pulmonology and Respiratory Medicine, Rumah Sakit Umum Pusat Persahabatan, Jakarta, IDN; 2 Psychiatry, Rumah Sakit Umum Pusat Persahabatan, Jakarta, IDN

**Keywords:** smoking tobacco, nicotine addiction, nicotine, abstinence, quit rate, smoking cessation, n-acetylcysteine (nac)

## Abstract

Introduction: Tobacco smoking remains a health concern, especially in developing countries. Nicotine is significantly linked to many cancers and even second-hand exposure. Hence, smoking can increase the risk of lung and heart disease. This makes quitting smoking important and challenging. Success tends to rise by achieving abstinence with assisted pharmacology. These treatments aim to reduce symptoms of nicotine withdrawal. This is a preclinical trial on glutamate modulator in N-acetylcysteine (NAC) as a new potential treatment for smoking cessation. It is based on the administration of NAC related to elevated levels of dopamine in the central nervous system to accomplish successful smoking cessation.

Aim: This study evaluated the efficacy and tolerability of NAC for smoking cessation. The primary outcome was abstinence rate and the secondary outcomes of the study were to assess carbon monoxide exhalation value (CO_exh_), the withdrawal symptoms, craving score, safety, and tolerability associated with the administration of NAC.

Methods: This is a randomized clinical trial. Eligible smokers were treated with NAC 2400 mg twice daily (BID) or placebo to obtain a potential effective abstinence rate. Subjects recruited from the smoking cessation clinic were screened for eligibility and were randomized to either the NAC or placebo group. The trial consisted of a four-week treatment phase and participants were evaluated each week with a brief counseling. Intention to treat data analysis was performed from 2018 to 2019. Smoking cessation status was verified by measuring the amount of carbon monoxide exhaled and by documenting their smoking habits. Adverse events (AEs) have also been observed on each visit.

Results: A total of 90 male smokers with a mean (SD) age of 38.7 (11) years were randomized into two groups to receive NAC (n=45) and placebo (n=45). The primary outcome revealed that the abstinence rate was significantly higher for the NAC group than the placebo group (37.7% vs 6.6%; p=0.02). These findings were supported by data comparison between the NAC group and placebo group of CO_exh_ (ppm) (9.59 ±7.4 vs 13,4 ±6.1; p=0.04) and cigarette consumption/week (10 vs 46; p <0.001), which were statistically significant. Comparison of withdrawal with the Minnesota Nicotine Withdrawal Score between the NAC group and the placebo group showed lower values (8 (1-31) vs 11 (0-43); p=0.178), respectively, even though not statistically significant. Compared to the placebo group, the craving score (6 (2-29) vs 12 (6-31); p=0.04) in the NAC group was significantly lower. The most common adverse event was mild gastrointestinal effects (28.9%) and arthralgia (2.2%). No serious adverse events were detected.

Conclusions: Despite a small sample size, the data demonstrate the potential benefits of NAC that may help elevate abstinence rates and promote successful smoking cessation pharmacotherapy. Comprehensive treatment combining pharmacologic therapy and counseling increases smoking cessation success rates. It is essential to conduct a randomized multicenter study with a large population to support a sustained abstinence rate using NAC.

## Introduction

Tobacco smoking poses a serious threat to health. Tobacco has been used worldwide throughout history, and the 20th century was the cigarette's golden age. Approximately 50% of people in industrialized nations smoked in 1950. Smoking was a socially acceptable behavior in every aspect of life [1]. Among the countries with the highest number of smokers, Indonesia is eighth, with a smoking rate of 37.60 percent, and has the highest rank among Southeast Asian Nations (ASEAN) [2]. The consumption of cigarettes in Indonesia has been consistently increasing over the past decade. Indonesia Ministry of Health (Kemenkes) recently launched the results of the 2021 Global Adult Tobacco Survey (GATS) which revealed an alarming increase in the number of adult smokers. The survey indicates that the number of adult smokers rose from 60.3 million in 2011 to 69.1 million in 2021, an increase of 8.8 million people [3,4].

The prevalence of active smokers among Indonesians aged 15 years and above in 2021 was 33.5%. The prevalence of ex-smokers is low, with around 6% of men and 1.7% of women having quit smoking [3]. Stopping smoking is difficult without support. Studies show that motivation and counseling improve success rates. Only 4% of tobacco users successfully quit without help. Although quit rates are low, combining new medication with counseling can increase success. Research suggests that smokers who receive both behavioral treatment and cessation medications are more likely to quit than those who receive minimal intervention [4,5]. 

Frequent exposure to nicotine causes the receptors to adapt to it, resulting in the development of tolerance to nicotine delivery. When a smoker quits smoking, they may experience nicotine withdrawal syndrome, which is characterized by a range of symptoms such as irritability, anxiety, increased appetite, and irritability. This makes it difficult for smokers to stop. Pharmacotherapies for smoking cessation should reduce withdrawal symptoms and block the reinforcing effects of nicotine without causing excessive adverse effects. Emerging preclinical evidence suggests that N-acetylcysteine (NAC) has potential as a pharmacotherapeutic agent due to its ability to modulate glutathione. It has been observed through various animal studies that NAC can regulate glutamate signaling and reduce the reinstatement of seeking behavior for drugs like heroin, cocaine, and nicotine [6-10]. NAC is a precursor of glutathione, an antioxidant that plays a crucial role in protecting cells from oxidative damage. Both of these compounds act as antioxidants and help reduce oxidative stress. The administration of NAC is related to elevated levels of glutamate and affects the exchange of dopamine release in the central nervous system. Maintaining a level of dopamine can stimulate the ability to reduce withdrawal and cravings in people determined to quit smoking [11,12].

NAC is a supplement that increases intracellular levels of glutathione, a major antioxidant, and modulates immune-inflammatory, oxidative, glutamatergic, and neurotrophic pathways. According to a randomized trial, taking 2.4 g/day of NAC for four weeks (compared to a placebo) resulted in a significant reduction in the number of cigarettes smoked by participants [12]. However, there was no observed reduction in craving, withdrawal symptoms, or level of carbon monoxide. There have been four small published pilot studies conducted to evaluate the effects of NAC on nicotine dependence [6,9,13,14]. One of these studies involved heavy smokers (N=22) who were treated for 3.5 days with NAC (3.6 g/day) or a placebo. The study found that participants who received NAC reported that their first cigarette was significantly less rewarding compared to those who received the placebo [6].

Pharmaceutical drugs with minimal side effects are highly desirable to overcome nicotine dependence. We thus conducted a clinical trial to assess the effectiveness of NAC in helping smokers quit in Asia while also observing withdrawal symptoms and craving scores.

## Materials and methods

This was a randomized, placebo-controlled study conducted at the Rumah Sakit Umum Pusat Persahabatan, Jakarta, Indonesia. The study was approved by the Ethics Committee of Rumah Sakit Umum Pusat Persahabatan, Jakarta (approval number: 32/KEPK-RSUPP/07/2017). Before participating in the trial, all enrolled participants provided written informed consent. The recruitment process started in January 2018 and ended in December 2018.

The following were the inclusion criteria for the study: (1) participants had to be males and 18 years or older, (2) they should have smoked at least 100 cigarettes in the past year, and have a carbon monoxide exhaled (CO_exh_) level of more than 6ppm, (3) they had to express willingness to participate in a smoking cessation program, and (4) they had to agree to follow the follow-up protocol. Exclusion criteria were as follows: (1) mental illness, including major depression, panic disorder, psychosis, or bipolar disorder that was diagnosed and clinical psychologists, and (2) Individuals with history or recently active gastrointestinal bleeding.

**Figure 1:**
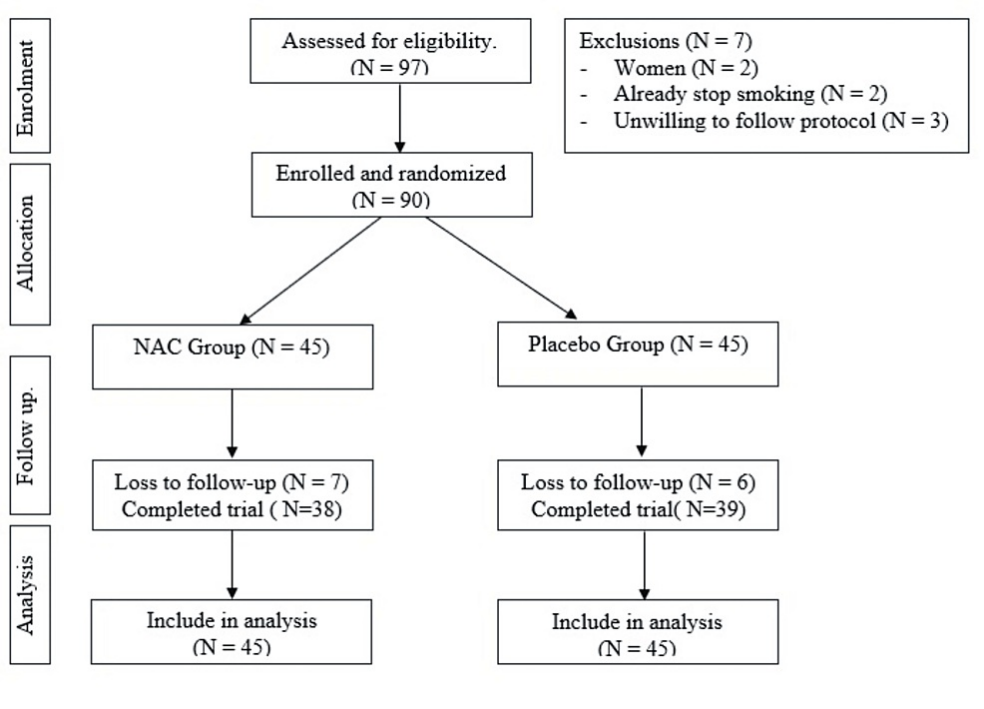
CONSORT diagram CONSORT: Consolidated Standards of Reporting Trials All lost to follow-up considered failed and included in analysis

The sample size calculation for this randomized controlled trial (RCT) was based on the abstinence rates from previous studies on smoking cessation [6,9,13,14]. To detect a significant difference, 30 subjects per group were necessary, with an estimated loss to follow-up of 20%. Therefore, the target number of participants was increased to a total of 90 (45 per study group). The test group got the active drug and the control group got a placebo. The first 97 smokers consecutively from the smoking cessation clinic at Rumah Sakit Umum Pusat Persahabatan were screened for eligibility. The majority of screening failures occurred because patients were unwilling to follow the protocol. The overall study design is summarized in Figure 1. 

Trial design

This study aimed to compare the effectiveness and safety of NAC 1200 mg, taken twice daily for four weeks, against a placebo for male smokers trying to quit smoking. The study lasted for a total of four weeks. Eligible smokers were invited to attend a baseline visit after screening. During the baseline visit, participants were screened for smoking status and underwent a medical history, physical examination, and CO_exh_ measurement. They were then randomized 1:1 to receive either NAC or placebo. Sociodemographic characteristics, smoking history, and motivation for quitting were self-reported and the following baseline measures were recorded: age first smoked, smoking duration, daily cigarettes, Brinkman Index, Fagerstrom Test for Nicotine Dependence (FTND), and CO_exh_. Participants were assigned follow-up assessments at weeks 2, 3, and 4.

After the first visit, smokers were invited to weekly visits for the next four weeks (visits 2-4). During each weekly visit, the physician provided brief individual counseling and assessed cigarette consumption, CO_exh_ measurement, withdrawal symptoms, craving score, and AEs. Participants received weekly blister packs with specific instructions enclosed. Adherence was assessed using medical diaries and pill counts. Regardless of their level of cigarette consumption, all patients were followed up. All participants received the study treatment and were included in the efficacy and safety analyses. We attempted to include all participants who were lost to follow-up and considered them as failed/still smoking in the analysis.

Outcomes measures

The primary efficacy endpoint was defined as the abstinence rate, as per previous NAC RCTs. The abstinence from smoking was determined by measuring CO_exh_ verified to be less than 6ppm during clinic visits. A piCO™ Smokerlyzer® (Bedfont Scientific Ltd, Maidstone, United Kingdom) was used for biochemical measurement. The secondary efficacy endpoints of the study were the withdrawal score, craving score, and AEs. The scoring was based on the Minnesota Nicotine Withdrawal Score (MNWS) and the craving score was measured by the Questionnaire on Smoking Urges (QSU) each week. All the questionnaires were on a quantitative scale. In short, the safety and endpoint of the study included information on the number of AEs that occurred between treatment randomization (visits 2-4).

Interventions

The pharmacy packaged NAC 1200 mg effervescence tablets and a matching placebo. The hospital pharmacy received secondary packaging of the treatment for blinding preparations. To ensure blinding, the labels from the containers of secondary packaging were removed, and the unlabeled study drugs were repacked in coded containers with identical appearances. Patients who were randomized to the NAC group were given 1200 mg twice a day for four weeks. All patients in both treatment groups received the same smoking cessation counseling throughout the trial. One-on-one counseling was provided at each visit by a trained physician.

Assessment

Assessments were conducted during the study visit, which included measurements and questionnaires: (1) CO_exh_, (2) FTND, a six-item questionnaire that classifies cigarette dependence into three levels; scores from 0-3 points indicated mild, 4-6 points indicated moderate, and 7-10 points indicated severe dependence, (3) QSU, which categorizes smoking urges into a 1-4 scale (ranging from strongly disagree to strongly agree), and (4) AEs.

Statistical analysis

The data collected were analyzed using IBM SPSS Statistics for Windows, Version 26.0 (Released 2019; IBM Corp., Armonk, New York, United States). Continuous variables were presented as mean ± standard deviation (SD), and categorical variables as percentages.

Baseline and demographic data were listed for all treatment groups. Summary statistics were provided for each treatment group. The normality of data distribution was tested using the Kolmogorov-Smirnov test. For normally distributed data, a t-test was used, while non-normally distributed data was analyzed using the Mann-Whitney U test. Differences in percentages between groups were evaluated using the Chi-square test (x^2^). Safety data were summarized with any events that were documented from the point of treatment initiation till the completion of four weeks of observation.

Despite the analysis of clinical and demographic features that could influence the outcome, this is not the main point of the study. We performed a prior selection of variables that were able to act as determinants, effect modifiers, or confounders of success in quitting sessions. The intention-to-treat (ITT) protocol was followed, meaning that all subjects were included in the analyses. All dropouts were considered as failures. All tests were two-sided and p <0.05 was considered to be significant. 

## Results

Ninety patients were randomized into two groups: NAC (N=45) group or placebo (N=45) groups. One group received NAC treatment, while the other group received a placebo. All participants were male with a mean age of 38.7±11 years of age. The mean age when they started smoking was 16.3±3.4 years. The mean duration of smoking was 22.8±12 years and the mean cigarettes smoked were 17.4±9.4 per day. Domination of the Brinkmann Index was moderate smokers (45.6%) with FTND moderate score (44.4%). The mean score for motivation to quit on a 10-point scale (1=not ready at all, 10=extremely ready) was 6.5±1.8. CO_exh_ level at baseline examination was 15.1±5.9 ppm. A total of 77 participants completed the treatment phase (85.5%; N=77); this was 84.5% in the NAC group (N=38) and 86.6% in the placebo group (N=39). Despite our best efforts to keep and support all randomized participants, 14% of participants discontinued treatment. Both groups were similar in terms of variables, and there was no significant difference between them (Table 1).

**Table 1:** Demographic and smoking characteristics. P< 0.05 was considered statistically significant NAC: N-acetylcysteine; CO_exh_: carbon monoxide exhaled Data presented as n (%) and mean ± SD

Characteristics	NAC Group (N=45)	Placebo Group (N=45)	Total (N=90)	p-value
Age (years), mean ± SD	41.4 ± 10.9	36.1 ± 10.9	38.7 ± 11	0.429
Age when first smoked (years), mean ± SD	17.2 ± 3.9	15.3 ± 2.4	16.3 ± 3.4	0.151
Smoking duration (years), mean ± SD	25.3 ± 13.5	20.5 ± 10	22.8 ± 12	0.08
Daily cigarettes, mean ± SD	18.2 ± 9	16.7 ± 9.8	17.4 ± 9.4	0.94
Brinkmann Index, n (%)				
Mild	17 (37.8)	18 (40.0)	35 (38.9)	0.515
Moderate	19 (42.2)	22 (48.9)	41 (45.6)	
Severe	9 (20)	5 (11.1)	14 (15.6)	
Fagestorm Test for Nicotine Dependence, n (%)				
Mild	20 (44.4)	13 (28.9)	33 (36.7)	0.459
Moderate	15 (33.3)	25 (55.6)	40 (44.4)	
Severe	10 (22.2)	7 (15.6)	17 (18.9)	
Motivation, mean ± SD	6.9 ± 1.8	6.2 ± 1.8	6.5 ± 1.8	0.7
Baseline CO_exh_ (ppm), mean ± SD	14.7 ± 6.6	15.4 ± 5.1	15.1 ± 5.9	0.319

Primary outcomes

The primary endpoint was the abstinence rate at the end of the four-week study (Table 2). The subjects reported their cigarette usage on weekly visits and were considered smokers until proven otherwise on the next visit through a COexh examination. The results showed that the abstinence rate was 37.7% in the NAC group and 6.6% in the placebo group. Additional correlation analyses between outcome measures showed a significant positive association from the free smoking day until the end of treatment. The non-parametric test for paired nominal data showed that the odds ratio (OR) was 5.66 (95%CI 1.78-17.9; p = 0.02), and the results were statistically significant.

**Table 2:** Abstinence rate Abstinence from smoking was defined as confirmed by carbon monoxide exhaled (CO_exh_) < 6 ppm; participants with CO_exh_  > 6 ppm were considered as smoking/failed. P-value <0.05 was considered significant. NAC: N-acetylcysteine; CO_exh_: carbon monoxide exhaled

Group	Abstinence at the end of four weeks		p-value
	Stopped, n (%)	Failed, n (%)	
NAC (N=45)	17 (37.7%)	28 (62.3%)	0.02
Placebo (N=45)	3 (6.7%)	42 (93.3%)	

Secondary outcomes

The secondary outcomes observed are presented in Table 3. Participants in the NAC group showed significant changes in CO_exh_ value mean (ppm) (9.59 ± 7.4 vs 13.42 ± 6.1; p=0.04) and cigarette consumption/day (10 vs 46; p <0,001). Meanwhile, other evaluations regarding the withdrawal and craving scores based on the questionnaire revealed that at the end of the study, only the craving score gave a significant value associated with quitting (6 vs 12; p=0.004). Among smokers, evaluation of each variable mostly demonstrated lower values in the NAC group compared to the placebo group in favor of smoking cessation.

**Table 3:** Secondary outcomes MNWS symptoms scale (ranging from 0 to 4), Craving score from Questionnaire on Smoking Urges (QSU) symptoms scale (ranging from 0-10). ^a^Analysis based on t-test; ^b^Analysis based on nonparametric test This data is not normal distribution. P-value <0.05 were considered statistically significant MNWS: Minnesota Withdrawal Score; NAC: N-acetylcysteine; CO_exh_: carbon monoxide exhaled

Variables	NAC Group	Placebo Group	p-value
CO_exh _value (ppm), mean ± SD	9.59 ± 7.4	13.42 ± 6.1	0.04^a^
Cigarette consumption (/week), median (min-max)	10 (0-112)	46 (0-168)	< 0.001^b^
MNWS score, median (min-max)	8 (1-31)	11 (0-43)	0.178
Craving score, median (min-max)	6 (2-29)	12 (6-31)	0.004

AEs

AE monitoring was carried out on a weekly basis. No serious adverse events or life-threatening incidents defined by the FDA were reported. The most common adverse event linked to gastrointestinal symptoms is likely or possibly associated with the study medication. Symptoms such as epigastric pain, heartburn, bloating, nausea, and belching were experienced by 13 participants (28.9%) in the NAC group and four participants (18.9%) in the placebo group. One participant (2.2%) in the NAC group reported arthralgia (Table 4). No other participant required termination. 

**Table 4:** Adverse events All instances are reported by one subject each. NAC: N-acetylcysteine

Symptoms	NAC group	Placebo group
	N = 14/45	N = 4/45
Gastrointestinal discomfort	13 (28.9%)	4 (18.9%)
Arthralgia	1 (2.2%)	0

## Discussion

This study presented evidence that NAC had an impact on substance use, particularly in tobacco smoking. As far as we know, this RCT was the first of its kind in an Asian country. One of the challenges in smoking control is the lack of accessibility to cessation support and almost no health insurance coverage for smoking treatments, including medications, counseling, or physician intervention. The results imply that significant changes in smoking behavior can occur through a combination of pharmacotherapy and counseling, rather than counseling alone. The administration of NAC was not associated with any differences in AEs between treatment groups, indicating a favorable safety profile. The results of previous NAC trials were also supported by these findings [6,11]. During the current trial, patients in the NAC group were found to be at least five times more likely to quit smoking compared to those in the placebo group. These findings were supported by measurements of COexh and self-reported daily cigarette consumption.

In our investigation on the factors that influence the rate of smoking abstinence, we observed that smokers who quit compared to those who failed to quit, regardless of whether they were in the NAC group or the placebo group, had a higher probability of achieving smoking abstinence. Several factors are substantially confounding such as amount of smoking, nicotine dependence, age of smoking, motivation, and lifetime smoking duration. It is difficult to disentangle the effect of these variables from each other without a well-planned study design. Moreover, individuals who smoked more cigarettes daily and had a higher level of nicotine dependence (measured by the FTND score) found it more difficult to quit smoking. it is important to take into account the individual's motivation level and lifetime smoking duration. Those with high motivation and shorter smoking duration have a higher likelihood of achieving successful cessation.

It is important to consider an individual's motivation level and lifetime smoking duration when attempting to quit smoking. This finding is not surprising. Those who have a high motivation level (score of >7) and a shorter smoking duration are more likely to successfully quit smoking. This is because smoking cessation medication can only help with relieving nicotine withdrawal symptoms and reducing reinforcement from smoking, but it cannot replace the need for smoking-related rituals. NAC is an amino acid precursor of glutathione, building part of glutamate level in cells. Both as antioxidants and rule in reducing oxidative stress [7,13]. Administration of NAC related to elevated levels of glutamate and affected exchange to dopamine release in the central nervous system. Maintaining a level of dopamine stimulates the ability to reduce withdrawal symptoms and cravings of people who are determined to stop smoking [8,14-16].

Some medications have been proven effective for stopping smoking in Europe and the United States, such as varenicline, bupropion, and NRT [5,11,17,18]. Although efforts to find other pharmacological alternatives are still ongoing, a study has gathered various clinical trials comparing drugs with placebo to the success of quitting smoking [9]. The results of studies show that quit rates on varenicline, bupropion, and NRT were statistically significant at 50.9%, 35.9%, and 32.2%, respectively [10,19]. NAC was found to be slightly comparable in average quit rate to those recommended by FDA drugs. The abstinence rate values may vary between 5-35%, depending on the type of pharmacology used, duration of administration, and intensity of companion modalities such as counseling or the administration of two types of drugs [10,19,20]. The trial duration varied in previous studies and ranged from four days [13] up to 24 weeks [6]. As a pilot research, the duration of the current study was four weeks due to the limitation of funding for the drugs. Although preliminary clinical findings have been promising, some evidence suggests that the efficacy of NAC may depend on its administration during a period of abstinence, rather than during use. This may explain the mixed results seen in clinical trials [21,22].

In the present study, it was found that in the first week, seven smokers in the NAC group were able to quit smoking, while only one smoker in the placebo group was able to do the same. In weeks 2-4, the number of successful quitters in the NAC group continued to increase, with a total of 17 smokers successfully quitting by the end of the study. It's important to note that all patients received weekly counseling and monitoring, and while some may have experienced slip-ups, we didn't collect data on the timing of these slip-ups. Non-pharmacological methods such as brief counseling and motivational techniques have been shown to have an impact on smoking cessation, even in the placebo group [5,23]. According to the trial by Wiratmoko et al., some smokers made successful smoking cessation even in the placebo group [5]. In all group treatments, smokers were given non-pharmacological methods such as brief counseling and motivational techniques that may have an impact in helping them stop smoking besides the pharmacotherapy effect. Combining both methods significantly improves the success rate. In the NAC group, a decrease in the number of cigarettes smoked was also followed by a decrease in CO_exh_, indicating consistent abstinence from smoking by the participants until the end of the observation period.

Our primary endpoint found that smokers who were able to quit smoking completely had a CO_exh_ < 6 ppm. Even though some smokers failed to stop, CO_exh_ in the NAC group was still considered lower than the placebo group. These findings correlate with the amount of cigarettes consumed in the NAC group compared to the placebo group. Several reductions of withdrawal and craving scores in the NAC group at the end of the four-week observation followed by decreased cigarette and CO_exh_ might show that NAC helps to restore regular glutamate homeostasis and signaling. Although some studies revealed no significant differences in withdrawal and craving substances, the complexity of other non-biological factors such as environmental and psychological aspects should also be considered. Despite the availability of therapeutics for the abstinence effect, AEs experienced revealed no serious effect and were considered tolerable [19,20]. Important brief counseling each week during observation ensures the smoker's compliance with NAC consumption. The study found that NAC was well-tolerated and had no serious side effects, but some participants did experience gastrointestinal symptoms like nausea, bloating, and vomiting. The number of dropouts was also lower than expected, at less than 20%.

Limitations of the study and recommendations

Our study has some limitations because it was not conducted across multiple centers, the treatment period was short, and the number of subjects was relatively small and unregistered. Further observation of the continued abstinence rate after the program would provide more data. Future studies with larger sample sizes, accompanied by biochemical markers, will allow for a deeper understanding of the multi-mechanism of action use of NAC. We have found evidence that NAC plays a role in abstinence for smoking cessation, which raises the intriguing possibility of using NAC as a new pharmacotherapy. This preliminary evidence provides support for the feasibility and safety of using NAC for the treatment of nicotine dependence.

## Conclusions

This placebo-controlled RCT was conducted in Indonesia and showed that NAC, along with behavioral support, is an effective and well-tolerated medication for quitting smoking. Further multi-centered randomized studies are needed to confirm the sustained quit rate of NAC.
